# Prognostic Value of Coronary CT Angiography-Derived Fractional Flow Reserve in Non-obstructive Coronary Artery Disease: A Prospective Multicenter Observational Study

**DOI:** 10.3389/fcvm.2021.778010

**Published:** 2022-01-31

**Authors:** Fan Zhou, Qian Chen, Xiao Luo, Wei Cao, Ziwen Li, Bo Zhang, U. Joseph Schoepf, Callum E. Gill, Lili Guo, Hong Gao, Qingyao Li, Yibing Shi, Tingting Tang, Xiaochen Liu, Honglin Wu, Dongqing Wang, Feng Xu, Dongsheng Jin, Sheng Huang, Haige Li, Changjie Pan, Hongmei Gu, Lixiang Xie, Ximing Wang, Jing Ye, Jianwei Jiang, Hanqing Zhao, Xiangming Fang, Yi Xu, Wei Xing, Xiaohu Li, Xindao Yin, Guang Ming Lu, Long Jiang Zhang

**Affiliations:** ^1^Department of Radiology, Jinling Hospital, Medical School of Nanjing University, Nanjing, China; ^2^Department of Radiology, Nanjing First Hospital, Nanjing Medical University, Nanjing, China; ^3^Department of Radiology, People's Hospital of Maanshan, Maanshan, China; ^4^Department of Radiology, The First People's Hospital of Xuzhou, Xuzhou, China; ^5^Department of Radiology, Lianyungang Clinical Medical College of Nanjing Medical University, Lianyungang, China; ^6^Department of Radiology, Taizhou People's Hospital, Taizhou, China; ^7^Division of Cardiovascular Imaging, Department of Radiology and Radiological Science, Medical University of South Carolina, Charleston, SC, United States; ^8^Department of Medical Imaging, The Affiliated Huai'an No.1 People's Hospital of Nanjing Medical University, Huai'an, China; ^9^Department of Medical Imaging, Qinhuai Medical Region of Jinling Hospital, Nanjing, China; ^10^Department of Radiology, Affiliated Hospital of Integrated Traditional Chinese and Western Medicine, Nanjing University of Chinese Medicine, Nanjing, China; ^11^Department of Diagnostic Radiology, Xuzhou Central Hospital, Xuzhou, China; ^12^Department of Diagnostic Radiology, Yancheng No.1 Hospital, Affiliated Hospital of Nantong University, Yancheng, China; ^13^Department of Medical Imaging, Hai'an City People's Hospital, Haian, China; ^14^Department of Radiology, The Affiliated Wujin Hospital of Jiangsu University, Changzhou, China; ^15^Department of Medical Imaging, Affiliated Hospital of Jiangsu University, Zhenjiang, China; ^16^Department of Medical Imaging, The Affiliated Suqian First People's Hospital of Nanjing Medical University, Suqian, China; ^17^Department of Radiology, Jiangsu Province Official Hospital, Jiangsu Jiankang Vocational College, Nanjing, China; ^18^Department of Radiology, The Second Affiliated Hospital of Nantong University, Nantong, China; ^19^Department of Medical Imaging, The Second Affiliated Hospital of Nanjing Medical University, Nanjing, China; ^20^Department of Radiology, The Affiliated Changzhou No.2 People's Hospital of Nanjing Medical University, Changzhou, China; ^21^Medical Imaging Center, Affiliated Hospital of Nantong University, Nantong, China; ^22^Department of Radiology, The Affiliated Hospital of Xuzhou Medical University, Xuzhou, China; ^23^Department of Radiology, The First Affiliated Hospital of Soochow University, Suzhou, China; ^24^Radiology Department, Subei People's Hospital of Jiangsu Province, Yangzhou, China; ^25^Department of Medical Imaging, Affiliated Hospital of Jiangnan University, Wuxi, China; ^26^Department of Medical Imaging, The Affiliated Huaihai Hospital of Xuzhou Medical University, Xuzhou, China; ^27^Department of Radiology, The Affiliated Wuxi People's Hospital of Nanjing Medical University, Wuxi, China; ^28^Department of Radiology, The First Affiliated Hospital of Nanjing Medical University, Nanjing, China; ^29^Department of Radiology, Third Affiliated Hospital of Soochow University, Soochow University, Changzhou, China; ^30^Department of Radiology, The First Affiliated Hospital of Anhui Medical University, Hefei, China

**Keywords:** non-obstructive coronary artery disease, coronary computed tomography angiography, fractional flow reserve, major adverse cardiovascular events, coronary plaque assessment

## Abstract

Coronary artery disease (CAD) is a major contributor to morbidity and mortality worldwide. Myocardial ischemia may occur in patients with normal or non-obstructive CAD on invasive coronary angiography (ICA). The comprehensive evaluation of coronary CT angiography (CCTA) integrated with fractional flow reserve derived from CCTA (CT-FFR) to CAD may be essential to improve the outcomes of patients with non-obstructive CAD. China CT-FFR Study-2 (ChiCTR2000031410) is a large-scale prospective, observational study in 29 medical centers in China. The primary purpose is to uncover the relationship between the CCTA findings (including CT-FFR) and the outcome of patients with non-obstructive CAD. At least 10,000 patients with non-obstructive CAD but without previous revascularization will be enrolled. A 5-year follow-up will be performed. The primary endpoint is the occurrence of major adverse cardiovascular events (MACE), including all-cause mortality, non-fatal myocardial infarct, unplanned revascularization, and hospitalization for unstable angina. Clinical characteristics, laboratory and imaging examination results will be collected to analyze their prognostic value.

## Introduction

Cardiovascular disease is a worldwide disease, but China is one of the countries with the heaviest disease burden (1). Since the 1980s, the burden of ischemic heart disease (IHD) in China has been increasing and has become more marked in the past 20 years (2). In 2013, IHD was reported to be the leading cause of death in six provinces, accounting for ~78% of the Chinese population (3). Moreover, IHD was ranked as the second leading cause of premature death in 2010 (2). The assessment and management of patients with coronary artery disease (CAD) have always focused on detecting and treating obstructive coronary artery stenosis (i.e., lumen obstruction of >50% [or >70%] by visual assessment) (4). However, the patients with CAD often are asymptomatic prior to myocardial infarction (MI), and coronary death with culprit lesions that are often non-obstructive before the events (5–7). The risk of cardiovascular events in patients with non-obstructive CAD is significantly higher than in patients without CAD (8, 9). Recently, the value of non-obstructive CAD and high-risk plaque (HRP) has been established (10).

Coronary CT angiography (CCTA) is a widely used non-invasive imaging modality to evaluate CAD and has demonstrated a high diagnostic performance in detecting obstructive and non-obstructive CAD on invasive coronary angiography (ICA) (11). Likewise, CCTA can also identify HRP seen on intravascular ultrasound or optical computed tomography (12). Coronary artery data and reporting system (CAD-RADS) has been reported to provide additional prognostic value beyond a coronary artery calcium score (CACS) and atherosclerotic cardiovascular disease (ASCVD) risk score (13). However, this reporting system does not consider the functional information of CAD, which is more important to determine the physiological nature of coronary stenoses than the anatomy (14). A previous study showed that 35% of non-obstructive CAD (diameter stenosis < 50%) was associated with ischemia (fractional flow reserve [FFR] ≤ 0.80), while 20% of lesions with >50% stenosis had no ischemia (15).

Coronary CT angiography-derived FFR (CT-FFR) has been reported to be a potential alternative to provide a physiological evaluation of the entire coronary artery tree, with high diagnostic accuracy in stable CAD patients compared with invasive FFR, the gold standard (16). Moreover, CCTA combined with selective CT-FFR is shown to be associated with a safety reduction of unnecessary ICA and relevant costs when compared with standard clinical practice (17). According to the ADVANCE (18) trial, patients with a negative CT-FFR have less revascularization and a trend toward lower major adverse cardiovascular events (MACE) and significantly lower MI or cardiovascular death. We hypothesize that integrating CCTA with CT-FFR will improve the recognition of subclinical plaques, optimize the risk stratification, and improve the prognosis of patients with non-obstructive CAD. Therefore, a large prospective study cohort from 29 medical centers in China (China CT-FFR Study-2) is designed to verify this hypothesis through a 5-year long-term follow-up.

The aims of this study are to (1) establish a large cohort of patients with non-obstructive CAD based on the Chinese population and conduct a long-term follow-up observation and (2) explore the predictive value of CT-FFR for MACE in patients with non-obstructive CAD beyond the clinical and anatomical factors.

## Methods and Analysis

### Study Design

This is a prospective, observational, multicenter registry study. The trial protocol and written informed consent forms have been reviewed and approved by the clinical trial ethics committee at Jinling Hospital, Medical School of Nanjing University (General Hospital of Eastern Theater Command), and by each center before the initiation of the investigation. All enrolled patients will provide written informed consent at admission for their data collection and utilization for future anonymous studies. The study has been registered on http://www.chictr.org.cn (ChiCTR2000031410). This study is partially supported by the National Key Research and Development Program of China (no. 2017YFC0113400), Jiangsu Provincial Key Research and Development Program (no. BE2020699), and Key Program of the National Natural Science Foundation of China (no. 81830057) for LJZ.

### Selection of Subjects

**Figure 1:**
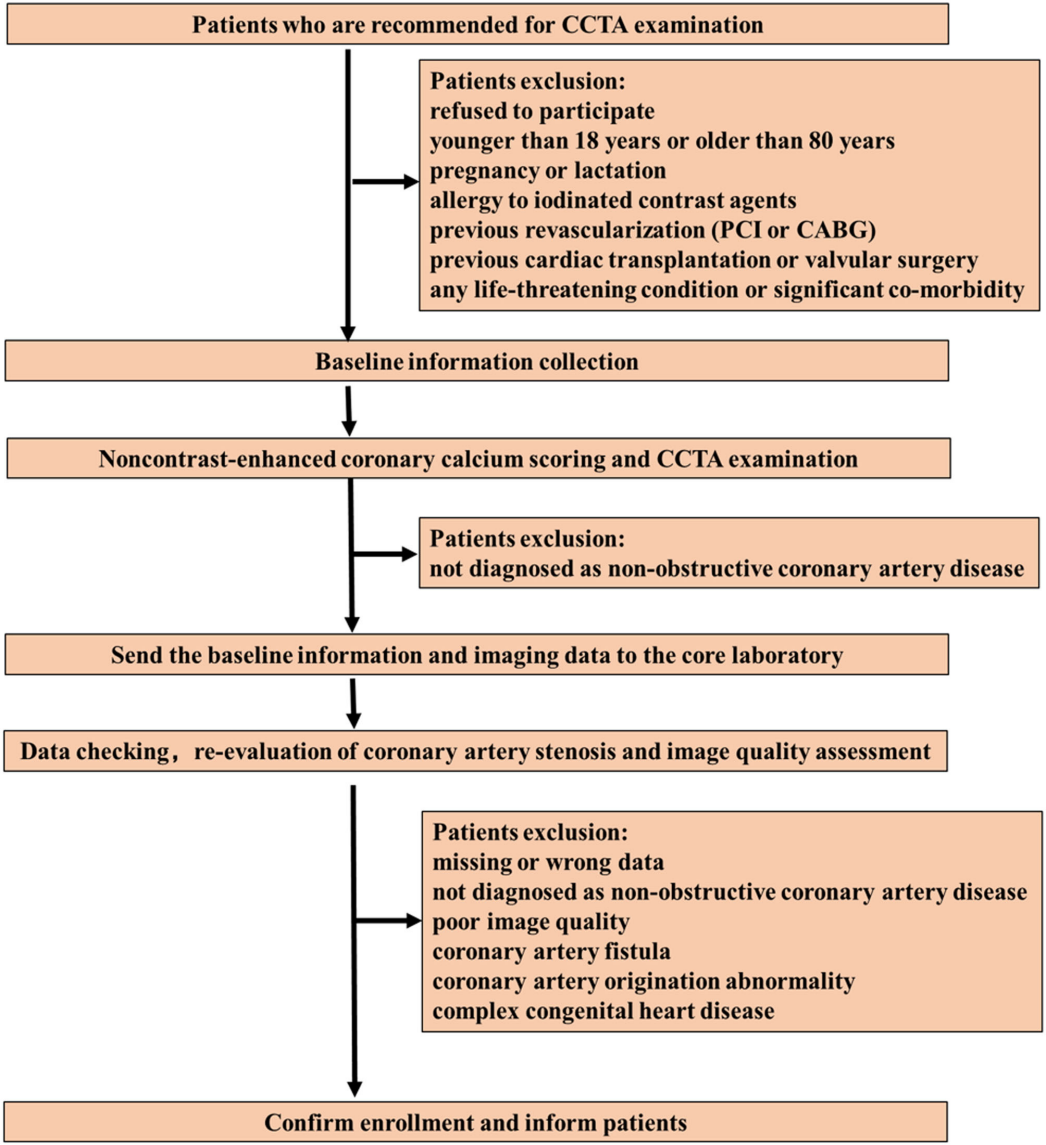
Patient enrollment flow chart. CCTA, coronary computed tomography angiography; PCI, percutaneous coronary intervention; CABG, coronary artery bypass grafting.

We aim to include ten thousand patients referred for CCTA examination by physicians for the evaluation of suspected or known CAD and subsequently diagnosed as non-obstructive CAD by CCTA (defined as a coronary artery stenosis [maximal stenosis] greater than or equal to 20% but <50% [for left main coronary artery] or greater than or equal to 20% but <70% [for any other epicardial coronary artery] in vessel segments ≥1.5 mm diameter) (19) but without previous revascularization (percutaneous coronary intervention [PCI] or coronary artery bypass grafting [CABG]). The enrollment process is shown in Figure 1.

### Inclusion Criteria

1) The patient must sign a written informed consent before any study procedures.
2) Non-obstructive coronary CAD by CCTA (a coronary artery stenosis [maximal stenosis] ≥20% but <50% [for left main coronary artery] or ≥20% but <70% [for any other epicardial coronary artery] in vessel segments ≥1.5 mm diameter).

### Exclusion Criteria

1) Refusal to participate.
2) <18 years or >80 years.
3) Pregnancy or lactation.
4) Allergy to iodinated contrast agents.
5) Previous revascularization (PCI or CABG).
6) Previous cardiac transplantation or valvular surgery.
7) Any life-threatening condition or severe co-morbidity.
8) Patients with poor image quality studies, unsuitable for further analysis.
9) Coronary artery fistula and anomalous origin.
10) Complex congenital heart disease.
11) Missing data.

During the initial screening stage (conducted by each center), any patients who meet any one of the above 1-7 exclusion criteria will be excluded. The final enrollment will be determined by the core laboratory (Jinling Hospital, Medical School of Nanjing University) after double-checking the data, re-evaluating coronary artery stenosis and image quality.

## Study Procedures

### Clinical Variables

Baseline clinical characteristics will be obtained through an electronic questionnaire for each participating patient at enrollment, including age, sex, height, weight, diabetes mellitus, hypertension, hyperlipidemia, smoking, drinking, positive CAD family history, clinical symptoms, all patient-reported examinations, medications, and surgeries associated with the cardiovascular disease within 6 months (Tables 1–5). Diabetes mellitus is defined as a history of diabetes mellitus diagnosed or treated by a physician, treated with oral antidiabetic therapy, or an admission fasting blood glucose level ≥126 mg/dl. Hypertension is defined as a history of high blood pressure diagnosed or treated by a physician, treated with antihypertensive therapy, or an admission blood pressure ≥140/90 mmHg. Dyslipidemia is defined as a history of hyperlipidemia diagnosed or treated by a physician, being in treatment with antihyperlipidemic drugs, or admission total cholesterol (TC) ≥200 mg/dl. Smoking/drinking is defined as current smoker/drinker or prior smoker/drinker within the last year. Positive CAD family history is defined as cardiac death or MI in first-degree relatives (<55 years in men or <65 years in women). Chest pain will be recorded and classified as typical angina, atypical angina and non-anginal chest pain according to the site, characteristics and duration of the chest discomfort and its relationship with exercise, and the inducing and relieving factors (20). Typical angina is considered to meet the following three characteristics: (a) constricting discomfort in the front of the chest or in the neck, jaw, shoulder, or arm; (b) induced by excessive physical activity; and (c) relieved by rest or nitrates within 5 min. Patients presenting with two of the aforementioned typical angina features are considered to have atypical angina. Patients presenting with only one or none of the typical angina characteristics are supposed to have non-anginal chest pain. Relevant medications will be recorded as statin, antiplatelet, beta-blockers, angiotensin-converting enzyme inhibitors (ACEI), angiotensin-receptor blockers (ARB), oral hypoglycemic agent, insulin, oral anticoagulation, calcium channel blocker (CCB), and diuretic. In addition, laboratory test results for C-reactive protein, triglycerides, TC, low-density lipoprotein cholesterol (LDL-C), high-density lipoprotein cholesterol (HDL-C), and cardiac enzyme level will be collected, if possible. The Framingham risk score (FRS) will be calculated for subjects with the complete information of age, sex, TC, HDL-C, systolic blood pressure, diabetes mellitus, antihypertensive medication, and smoking status (21). Meanwhile, the Multi-Ethnic Study of Atherosclerosis (MESA) score will also be calculated using age, gender, race/ethnicity, diabetes mellitus, smoking status, TC, HDL-C, antihyperlipidemic drugs, and MI family history (22).

**Table 1:** Baseline demographic information.

Name: _________________ ID number:______________
Birthday:_________________ Sex: □Male □Female
Height: cm Weight: kg
Telephone number (participant):_________________
Telephone number (first relatives):_________________
Relationship between relatives and participant:_______________

*ID, Identity document; cm, centimeter; kg, kilogram*.

**Table 2:** Baseline clinical information.

1. Hypertension: □Yes □No (meet one of the following three factors)
□systolic blood pressure ≥140 mmHg
□diastolic blood pressure ≥90 mmHg
□being treated with antihypertensive therapy
2. Diabetes mellitus: □Yes □No (meet one of the following two factors)
□fasting blood glucose level ≥126mg/dl
□being treated with oral antidiabetic therapy
3. Dyslipidemia: □Yes □No (meet one of the following two factors)
□total cholesterol ≥200mg/dl
□being treated with antihyperlipidemic drugs
4. Smoking: □Yes □No (meet one of the following two factors)
□current smoker
□previous smoker within the last year
5. Drinking: □Yes □No (meet one of the following two factors)
□current drinker
□previous drinker within the last year
6. Positive family history: □Yes □No (meet one of the following two factors)
□cardiac death or MI in first-degree relatives younger than 55 years in men
□cardiac death or MI in first-degree relatives younger than 65 years in women
7. Clinical symptoms:
□chest pain: □in the front of the chest or in the neck, jaw, shoulder, or arm
□precipitated by physical exertion
□relieved by rest or nitrates within 5 min
□palpitation □dyspnea □syncope □other

*MI, myocardial infarction*.

**Table 3:** Baseline medications information.

Whether there is a history of cardiovascular disease-related drug use within 6 months before the examination: □Yes □No (If yes, continue to fill in the content below)
□statin: dose:________ duration: from_____to
□antiplatelet: dose:________duration: from_____to
□beta-blocker: dose:________ duration: from_____to
□ACEI: dose:________duration: from_____to
□ARB: dose:________ duration: from_____to
□CCB: dose:________duration: from_____to
□diuretic: dose:________ duration: from_____to
□oral hypoglycemic agents: dose:________ duration: from_____to
□insulin: dose:________ duration: from_____to
□oral anticoagulation: dose:________duration: from_____to
□other: dose:________ duration: from_____to

*ACEI, angiotensin converting enzyme inhibitor; ARB, angiotensin-receptor blocker; CCB, calcium channel blocker*.

**Table 4:** Baseline surgical information.

Whether there is a history of cardiovascular disease-related surgeries within 6 months before the examination: □Yes □No (If yes, continue to fill in the content below)
1. Surgical method:
Surgical date:
Postoperative diagnosis:
2. Surgical method:
Surgical time:
Postoperative diagnosis:
3. Surgical method:
Surgical time:
Postoperative diagnosis:
4. Surgical method:
Surgical time:
Postoperative diagnosis:
……

**Table 5:** Baseline imaging tests information.

Whether there is a history of cardiovascular disease-related imaging examinations before the examination: □Yes □No (If yes, continue to fill in the content below)
1. Examination method: □CT □MRI □OCT □PET □SPECT □other
time:__________ region:
diagnosis:
2. Examination method: □CT □MRI □OCT □PET □SPECT □other
time:__________ region:
diagnosis:
3. Examination method: □CT □MRI □OCT □PET □SPECT □other
time:__________ region:
diagnosis:
4. Examination method: □CT □MRI □OCT □PET □SPECT □other
time:__________ region:
diagnosis:
5. Examination method: □CT □MRI □OCT □PET □SPECT □other
time:__________ region:
diagnosis:
……

### CCTA Scanning Protocols

All CCTA acquisitions will be performed in each medical center using CT scanners with ≥64 detector rows. A non-contrast enhanced coronary calcium scoring study will be obtained before the CCTA examination. The specific scanning protocols are consistent with the routine clinical practice of CCTA in each medical center. Nitroglycerin and beta-blockers will be administered according to the standard practice of each center. Baseline scanning characteristics will be obtained through an electronic questionnaire for each participating patient after CCTA examination, such as scanning equipment, scanning protocols, the name, concentration, dosage and injection rate of iodinated contrast agent, average heart rate during scanning, and the usage of nitroglycerin and beta-blockers. All the Digital Imaging and Communications in Medicine (DICOM) files will be transferred to the core laboratory online or offline for image quality and coronary stenosis assessment after the initial screening in each center. After double-checking the data, subjects who meet the inclusion criteria will be finally enrolled and informed by the core laboratory.

### Image Quality Analysis

The CCTA images will be evaluated in consensus by two cardiovascular radiologists (FZ and CXT, with 5 and 8 years of experience in CCTA interpretation) at the core laboratory. Image quality assessment will be performed using a four-point Likert scale as follows: 4 = excellent (clear delineation of the coronary arteries, no motion artifacts, and slight noise); 3 = good (mild blurring and minor motion artifacts); 2 = acceptable (moderate blurring and mild motion artifacts or minor discontinuity); and 1 = non-diagnostic (severe motion artifacts or discontinuity made the coronary artery structures not differentiable). Patients with poor image quality (score = 1) unsuitable for further analysis will be excluded (23).

### Coronary Plaque Evaluation

The coronary artery is divided into 17 segments according to the American Heart Association (AHA) classification (24). The right coronary artery (RCA) includes segments 1, 2, 3, 4, and 16. The left main (LM) and left anterior descending (LAD) include segments 5, 6, 7, 8, 9, and 10. The left circumflex (LCX) includes segments 11, 12, 13, 14, and 15. Coronary plaques will be identified in segments with a diameter ≥1.5 mm. Lesions located in the major epicardial coronary arteries will be classified as proximal (segments 1, 6, and 11), middle (segments 2 and 7), and distal (segments 3, 8, and 13).

#### Evaluation of the Coronary Plaque Calcification

To quantify coronary plaque calcification, the Agatston score (AS) will be calculated for each patient by multiplying the calcified lesion area by the maximum density of a single target lesion in this area using a semiautomated software (SyngoVia, Siemens Healthineers, Forchheim, Germany) (25). Based on the AS, patients will be divided into four groups: 0, 1–99, 100–299, and ≥300 (26). In addition, we will evaluate the degree of plaque calcification of the most severe lesion using the arc of calcification in the short axis. Accordingly, the degree of target lesion calcification is defined as: (1) no calcification (no calcification present); (2) mild (<90°arc calcification); (3) moderate (90–180°arc calcification); (4) severe (180–270°arc calcification); or (5) very severe (270–360°arc calcification) (27).

#### Quantitative Evaluation of the Coronary Plaque

Quantitative plaque analysis will be performed by a group of observers using a semiautomated software (QAngioCT, Medics, Leiden, The Netherlands). The following plaque parameters will be assessed: %DS (degree of lumen stenosis calculated at the site of maximal stenosis, defined as 100% × [reference diameter-minimal lumen diameter, MLD]/reference diameter); plaque length; MLD and minimal lumen area (MLA); plaque volume (defined as vessel volume minus lumen volume), such as the total volume, the volume of calcification components (>350 HU), lipid components (<30 HU), fiber-fatty components (30–130 HU) and fibrous components (131–350 HU) (28); plaque burden (PB) (defined as 100% × [plaque volume/vessel volume]); and the remodeling index (RI) (automatically calculated), calculated as the diameter, i.e., both plaque and vessel lumen at the site of maximal stenosis divided by the mean diameter of the proximal and distal reference site. Plaque volume and burden will be summed on a per-segment, per-vessel, and per-patient level. According to the RI, lesions will be categorized into three groups as follows: positive remodeling (PR; RI > 1.0), negative remodeling (RI < 0.88), and intermediate remodeling (0.88 ≤ RI ≤ 1.0).

On a per-lesion basis, CAD-RADS categories are defined as follows: CAD-RADS 1 (1–24% stenosis or present coronary plaque without apparent stenosis), CAD-RADS 2 (25–49% stenosis), and CAD-RADS 3 (50–69% stenosis). On a per-segment/vessel/patient basis, CAD-RADS categories will be evaluated based on the maximal stenosis. According to the AHA classification, the number of segments ≥1.5 mm diameter with any calcified, non-calcified, or mixed plaque will be recorded, regardless of the degree of stenosis, for the calculation of segment involvement score (SIS). The maximum SIS value that can be derived is 17 for each patient. A comprehensive CTA risk score will be calculated using an online calculator (http://18.224.14.19/calcApp/) according to the previous study (29). The CCTA risk score is derived from CONFIRM registry, and the calculated content includes the presence, location, extent, severity, and composition of CAD.

#### Qualitative Evaluation of the Coronary Plaque

According to previous studies, four signs of coronary artery plaques on CCTA will be recorded as HRP features, namely, low-density plaque (LAP), PR, punctate calcification (SC), and napkin ring sign (NRS) (26). LAP is defined as the plaque component with an attenuation of <30 HU. RI > 1 indicates PR. SC is defined as any discrete calcification less than or equal to 3 mm in length and occupying less than or equal to 90° arc when viewed in the short axis. NRS is recognized as a central low attenuation plaque with a peripheral rim of higher attenuation of the non-calcified plaque.

### CT-FFR Measurements

All CT-FFR values will be calculated by using an automated software (“Shukun-FFR” software from Shukun [Beijing] Technology Co., Ltd). The “Shukun-FFR” software consists of two main components, the coronary arteries segmentation model and the computational fluid dynamics (CFD) simulation model. Specifically, a modified V-Net is first used to to segment coronary arteries from the CCTA image firstly (30). Then, we adopt prior knowledge to name vascular branches based on additional anatomical rules, using previous vessel segmentation results as input. The final reduced-order CFD model is applied to compute the flow and pressure of blood and calculate CT-FFR values automatically for all points along coronary arteries. CT-FFR values will be determined for coronary arteries ≥1.5 mm in diameter in the core laboratory by two cardiovascular radiologists (observers A and B). The automatically identified coronary plaques and the degree of lumen stenosis will be verified by another well-experienced observer. Observer A will carry out measurements two times separately (recorded as A1, A2). The time interval between the two measurements is 2 weeks to eliminate the effect of memory; observer B will measure one time (recorded as B). CT-FFR values will be measured at the proximal, distal, and 20 mm distal to the stenosis and the end of the target vessel (at least 1.5 mm diameter). CT-FFR ≤ 0.80 indicates the hemodynamic significance of coronary stenosis. Patients with CT-FFR ≤ 0.80, which is 20 mm distal to the stenosis, are deemed to have lesion-specific ischemia. Patients without lesion-specific ischemia but with CT-FFR ≤ 0.80 at the end of the target vessel are deemed to have distal vessel ischemia.

### Endpoints and Definition

Outcomes will be ascertained by phone contact with the patient (or family member if needed), reviewing medical records or consulting with the attending physicians, if possible, and adjudicated blindly by an independent clinical event committee according to ACC/AHA standards and the fourth universal definition of MI by reviewing symptoms, cardiac laboratory biomarker data, electrocardiogram results, and cardiovascular imaging findings (31, 32).

### Primary Endpoints

The primary endpoint is the occurrence of MACE at follow-up, defined as a composite of all-cause death, non-fatal MI, unplanned revascularization (at least 60 days after the CCTA examination), and hospitalization for unstable angina. Death cases consist of cardiovascular death, non-cardiovascular death, and undetermined cause of death. Cardiovascular death includes death attributable to sudden cardiac death, acute MI, heart failure, cardiovascular bleeding, stroke, cardiovascular procedure, and other cardiovascular causes, such as pulmonary embolism. When a definite non-cardiovascular cause is documented, a cardiovascular reason for death could be excluded. Non-fatal MI consists of ST-segment elevation myocardial infarction (STEMI) and non–STEMI. Unstable angina is defined as the symptoms of ischemic chest pain at rest, and the final diagnosis is myocardial ischemia with objective evidence but without elevation of cardiac biomarkers. For the primary endpoints, the first occurrence timepiont of any one of the above events will be recorded. The follow-up duration is recorded as the interval from baseline CCTA examination to the day of follow-up.

### Secondary Endpoints

Secondary endpoints include cardiovascular death, atrial fibrillation, non-fatal MI, non-fatal stroke, unplanned revascularization, hospitalization for unstable angina, medication adjustment, invasive or non-invasive cardiovascular tests, cumulative radiation exposure from all cardiovascular tests, and total cardiac costs. Medication adjustment includes the increase or decrease of the drug dose and the new addition or discontinuation of medications. For the secondary endpoints, the time of the occurrence for any one of the above events should be recorded.

### Follow-Up

Telephone follow-up will be performed for each enrollee annually by the follow-up team of each medical center until the completion of the 5-year follow-up. The follow-up team in each center includes at least one nurse, one cardiovascular radiologist, and one cardiovascular physician. The follow-up team will first contact the patient via a phone call. If it is unsuccessful, the personal contact will be called for updated information. After documentation of three unsuccessful attempts (on three different days over 1 month) by the follow-up team, a subject will be considered lost to telephone follow-up. If the outcome of this subject cannot be traced through the medical record system, the subject will eventually be deemed to be lost to follow-up. The follow-up team of the core laboratory will randomly select 10% of the subjects from each other medical center for review to confirm the authenticity of the follow-up data.

### Data Collection and Management

Data management and coordination will be performed by the core laboratory. Study investigators in each medical center will collect all text data regarding baseline clinical characteristics, laboratory results, medical/interventional treatments and outcomes and enter them into a web-based electronic case report form. All imaging data will be transmitted to the core laboratory in an anonymous form. A reference number will be assigned to every single enrolled patient. The final China CT-FFR Study-2 database, including text and imaging data, will be constructed from the individual and valid databases, which will be provided by each participating center. Centers providing forms with more than 5% of missing values will be excluded from the final China CT-FFR Study-2 database.

### Trial Status

The study was started in November 2019, and initially included 24 medical centers from all 13 regions of Jiangsu Province (Table 6). Due to the COVID-19 pandemic (from February 2020 to July 2020), study progress was significantly delayed. In August 2020, another three medical centers from Jiangsu Province and two from Anhui Province joined the study, with the agreement of the members of the Data Safety and Monitoring Board and a common end date of December 30, 2021 was defined. The number of target subjects for each center is determined based on the number of daily CCTA examinations and each centers requests.

**Table 6:** Detail information of the medical centers.

**No**.	**Centers**	**Principal Investigator (PI)**	**Target number of subjects**
1	Jinling hospital	Long Jiang Zhang	1,000
2	Nanjing first hospital	Xindao Yin	1,000
3	Taizhou people's hospital	Bo Zhang	500
4	The first affiliated hospital of nanjing medical university	Yi Xu	500
5	The affiliated wuxi people's hospital of nanjing medical university	Xiangming Fang	500
6	Affiliated hospital of jiangnan university	Jianwei Jiang	500
7	Subei people's hospital of jiangsu province	Jing Ye	500
8	The first affiliated hospital of soochow university	Ximing Wang	500
9	The affiliated hospital of xuzhou medical university	Lixiang Xie	500
10	The first affiliated hospital of anhui medical university^*^	Xiaohu Li	500
11	Affiliated hospital of nantong university	Hongmei Gu	300
12	The affiliated huai'an No.1 people's hospital of nanjing medical university	Lili Guo	300
13	Third affiliated hospital of soochow university	Wei Xing	300
14	The affiliated changzhou No.2 people's hospital of nanjing medical university	Changjie Pan	300
15	The second affiliated hospital of nanjing medical university	Haige Li	200
16	The second affiliated hospital of nantong university	Sheng Huang	200
17	The first affiliated hospital of kangda college nanjing medical university	Ying Zhou	200
18	Jiangsu province official hospital	Dongsheng Jin	200
19	Affiliated hospital of jiangsu university	Dongqing Wang	200
20	The affiliated suqian first people's hospital of nanjing medical university	Feng Xu	200
21	The affiliated wujin hospital of jiangsu university	Honglin Wu	200
22	Qinhuai medical region of jinling hospital	Hong Gao	200
23	Hai'an city people's hospital	Ping Wu	200
24	Yancheng No.1 hospital	Dingyou Lu	200
25	The huaihai affiliated hospital of xuzhou medical university	Hanqing Zhao	200
26	Xuzhou central hospital^*^	Yibing Shi	200
27	The first people's hospital of xuzhou^*^	Wei Cao	200
28	Affiliated Hospital of Integrated Traditional Chinese and Western Medicine^*^	Zongjun Zhang	100
29	People' hospital of maanshan^*^	Xiao Luo	100

* *Medical centers joined in August 2020*.

### Data Analysis

According to the Kolmogorov-Smirnov test, continuous data will be categorized as normally or non-normally distributed data. Mean ± SD will be used to express the quantitative variables for uniformity of presentation, regardless of the distribution. Independent sample *t*-tests, paired *t*-tests, or ANOVA tests will be conducted for the comparison of normally distributed continuous data, as appropriate. The difference analysis of non-normally distributed data will be performed by using the non-parametric test. Categorical variables will be expressed as presented as numbers and frequencies or percentages. Pearson's chi-squared test or the likelihood ratio will be used to compare the differences of categorical data between groups, as appropriate. Univariate and multivariate Cox proportional hazard models will be performed to calculate the hazard ratio (HR) with a 95% CI and to estimate the association between variables and the hazards of MACE. Kaplan-Meier plots with the log-rank tests will be generated to estimate cumulative rates of MACE. The receiver operating characteristics (ROC) curve analysis will be performed to determine the optimal cut-off values of quantitative data. The area under the curve (AUC) for each model will be compared. The patient's annual total cardiac cost will be compared using the non-parametric Wilcoxon rank-sum test or a two-sample *t*-test. Intraclass correlation coefficient (ICC) and Spearman correlation coefficient will be used to assess the inter- and intra-observer agreement. Considering the selection bias and potential heterogeneity between 29 centers, a center effect analysis will be performed. If there is a central effect, a stratified analysis and sensitivity analysis will be performed, or it will be adjusted as a random factor. A *p* ≤ 0.05 is considered statistically significant.

## Discussion

Coronary CT angiography has been recommended as the first-choice imaging modality for patients with stable CAD. In addition to the unique ability for non-invasive qualitative and quantitative evaluation of coronary atherosclerotic plaque, CCTA can provide functional information without the increased burden of radiation exposure and administration of stress agents. Compared with conventional CCTA alone, it has been demonstrated that CT-FFR had improved discrimination of ischemia (33). The Prospective Multicenter Imaging Study for Evaluation of Chest Pain (PROMISE) trial demonstrated that the 9-month costs of patients with stable angina in the CT-FFR group were slightly higher than that in the routine care group, but the difference was not significant (*p* = 0.10) (34). The inconsistent conclusions about the socioeconomic effects of CT-FFR with previous studies may be due to the fact that only 31% of the patients in the CT-FFR group have undergone CT-FFR examinations in this study (35, 36). In addition, all patients have stable angina, but CT-FFR is believed to minimize unnecessary care and resources for patients with mild stenosis and equivocal stenosis, which highlights the significance of CT-FFR for patients with non-obstructive CAD (37). The previous study showed that among symptomatic patients who underwent CCTA, only a few patients were diagnosed with obstructive CAD (8). However, it is reported that about 10% of total MI is caused by non-obstructive CAD (MINOCA), and 18.7% of MINOCA patients will have MACE after 1-year of follow-up (38). Although several CCTA-based scoring systems have been proven to have prognostic value, the functional information was not included in these studies (13, 29, 39). The China CT-FFR Study-2 is designed to investigate the potential ability of CT-FFR to improve the risk stratification of patients with non-obstructive CAD beyond the clinical and morphological information using large-scale data with long-term follow-up. The baseline clinical information can be used to explore its relationship with the CAD. The baseline CCTA findings will provide the data on the morbidity of non-obstructive CAD. The longitudinal follow-up data will uncover the association between CT-FFR and HRP identified on baseline CCTA and downstream risk of MACE. Further, this observational registry will be valuable for the design of future randomized controlled trials.

Despite its strengths, this study has some limitations. First, this is a large observational registry, which has inherent limitations, such as selection bias and potential heterogeneity between centers. Second, most of the medical centers are from the same province, resulting in a lack of external verification. Third, there is no uniform scanning protocol for baseline CCTA, the scanning characteristics should be adjusted during subsequent data analysis. The scanning protocols of each center will be recorded for the central effect analysis. If there is a central effect, a stratified analysis and sensitivity analysis will be performed, or it will be adjusted as a random factor. Fourth, no blood or other biological samples will be collected at the baseline of this study, which may cause some critical information to be missed.

## Conclusion

The China CT-FFR Study-2 is a prospective, observational, multicenter registry study. At least 10,000 patients with non-obstructive CAD will be enrolled from 29 medical centers in China. Baseline clinical information and CCTA findings (such as CT-FFR) will be collected. A 5-year follow-up will be performed by phone contact to ascertain the outcomes of patients. To the best of our knowledge, this is the largest prospective observable study to explore the relationship between CT-FFR and prognosis in China. Based on the excellent performance of CT-FFR for diagnosing myocardial ischemia and its predictive value for future risk of MACE, we believe that the China CT-FFR Study-2 will be helpful to provide unique risk stratification of patients with non-obstructive CAD.

## Ethics Statement

The studies involving human participants were reviewed and approved by Clinical Trial Ethics Committee of General Hospital of Eastern Theater Command. The patients/participants provided their written informed consent to participate in this study.

## Author Contributions

LJZ and GML contributed to conception and design of the study. FZ was a major contributor in writing the manuscript. FZ, QC, XLu, WC, ZL, and BZ made the equal contribution to this study. QC, ZL, QL, TT, and XLiu contributed to data collection. LJZ, GML, XY, XLi, XLu, WC, BZ, LG, HGa, YS, HW, DW, FX, DJ, SH, HL, CP, HGu, LX, XW, JY, JJ, HZ, XF, YX, and WX organized the database. UJS and CEG contributed to the language revisions. All authors contributed to manuscript revision, read, and approved the submitted version.

## Funding

This study was funded by the National Key Research and Development Program of China (no. 2017YFC0113400), the Jiangsu Provincial Key Research and Development Program (no. BE2020699), and the Key Program of the National Natural Science Foundation of China (no. 81830057) for LJZ. XLi was funded by the National Natural Science Foundation of China (no. 82071897).

## Conflict of Interest

UJS is a consultant for and/or receives research support from Bayer, Bracco, Elucid Bio, Guerbet, HeartFlow, Keya Medical, and Siemens Healthineers. The remaining authors declare that the research was conducted in the absence of any commercial or financial relationships that could be construed as a potential conflict of interest.

## Publisher's Note

All claims expressed in this article are solely those of the authors and do not necessarily represent those of their affiliated organizations, or those of the publisher, the editors and the reviewers. Any product that may be evaluated in this article, or claim that may be made by its manufacturer, is not guaranteed or endorsed by the publisher.
